# Attention allocation to negatively-valenced stimuli in PTSD is associated with reward-related neural pathways

**DOI:** 10.1017/S003329172200157X

**Published:** 2023-07

**Authors:** Benjamin Suarez-Jimenez, Amit Lazarov, Xi Zhu, Daniel S. Pine, Yair Bar-Haim, Yuval Neria

**Affiliations:** 1 Department of Neuroscience, The Del Monte Institute for Neuroscience, University of Rochester School of Medicine and Dentistry, Rochester, NY, USA; 2 School of Psychological Sciences, Tel-Aviv University, Tel-Aviv, Israel; 3 Department of Psychiatry, Columbia University Irving Medical Center, New York, NY, USA; 4 Department of Psychiatry, Columbia University Irving Medical Center and New York State Psychiatric Institute, New York, NY, USA; 5 Section on Developmental Affective Neuroscience, National Institute of Mental Health, Bethesda, MD, USA; 6 School of Psychological Sciences and Sagol School of Neuroscience, Tel Aviv University, Tel Aviv, Israel; 7 Department of Psychiatry, Columbia University Medical Center and New York State Psychiatric Institute, New York, NY, USA

**Keywords:** Attention allocation, eye-tracking, PTSD, resting-state functional connectivity (rsFC), reward functioning, trauma-exposure

## Abstract

**Background:**

In a recent eye-tracking study we found a differential dwell time pattern for negatively-valenced and neutral faces among patients with posttraumatic stress disorder (PTSD), trauma-exposed healthy control (TEHCs), and healthy control (HC) participants. Here, we explored whether these group differences relate to resting-state functional connectivity (rsFC) patterns of brain areas previously linked to both attention processes and PTSD. These encompass the amygdala, dorsal anterior cingulate cortex (dACC), dorsolateral prefrontal cortex (dlPFC), ventrolateral prefrontal cortex (vlPFC), and nucleus accumbens (NAcc).

**Methods:**

Ten minutes magnetic resonance imaging rsFC scans were recorded in 17 PTSD patients, 21 TEHCs, and 16 HCs. Participants then completed a free-viewing eye-tracking task assessing attention allocation outside the scanner. Dwell time on negatively-valenced stimuli (DT%) were assessed relative to functional connectivity in the aforementioned seed regions of interest (amygdala, dACC, dlPFC, vlPFC, and NAcc) to whole-brain voxel-wise rsFC.

**Results:**

As previously reported, group differences occurred in attention allocation to negative-valence stimuli, with longer dwell time on negatively valence stimuli in the PTSD and TEHC groups than the HC group. Higher DT% correlated with weaker NAcc-orbitofrontal cortex (OFC) connectivity in patients with PTSD. Conversely, a positive association emerged in the HC group between DT% and NAcc-OFC connectivity.

**Conclusions:**

While exploratory in nature, present findings may suggest that reward-related brain areas are involved in disengaging attention from negative-valenced stimuli, and possibly in regulating ensuing negative emotions.

Cognitive models of posttraumatic stress disorder (PTSD) implicate biased attention to threat-related information in the disorder (Aupperle, Melrose, Stein, & Paulus, 2012; Brewin & Holmes, 2003; Buckley, Blanchard, & Neill, 2000; Chemtob, Roitblat, Hamada, Carlson, & Twentyman, 1988; Ehlers & Clark, 2000; Foa & Rothbaum, 1998; Foa, Steketee, & Rothbaum, 1989; Litz & Keane, 1989). According to these models, the attentional system of patients with PTSD is biased toward threat-related information (Aupperle et al., 2012; Chemtob et al., 1988; Ehlers & Clark, 2000; Foa et al., 1989; Foa & Rothbaum, 1998; Litz & Keane, 1989). Some of the strongest data on attention biases arise from studies using eye-tracking methodology, which consistently find more sustained eye-gaze on threat-related information (i.e. sustained attention) in participants with PTSD (for a review see Lazarov et al., 2019a, 2019b; more recently Lazarov et al., 2021b; Mekawi et al., 2020; Powers et al., 2019). Despite the strength and consistency of this finding, few studies map the neural correlates of sustained eye-gaze in PTSD. The current study used resting-state functional magnetic resonance imaging to generate preliminary data on such neural correlates.

Recently, we have adapted an established eye-tracking free-viewing task for research in PTSD. The task assesses attention allocation within matrices containing multiple competing negative-valence and neutral faces (Lazarov et al., 2021b). In this study, patients with PTSD, trauma-exposed healthy control (TEHC) participants, and non-exposed healthy control (HC) participants freely viewed visual displays comprised of matrices with 16 faces, half negatively-valenced (i.e. anger, fear, or sad expressions) and half neutral. To maximize the relevance of facial stimuli as trauma-related cues, participants in the two trauma-exposed groups were included only if they met DSM-5 criterion A for a traumatic event of an interpersonal nature (Forbes et al., 2014; Kelley, Weathers, McDevitt-Murphy, Eakin, & Flood, 2009; Kessler & Üstün, 2004), as prior research has shown the emotional effect of viewing negative facial expressions in PTSD (Armony, Corbo, Clement, & Brunei, 2005; Rauch et al., 2000), and more specifically in those with a history of an interpersonal trauma (Fonzo et al., 2010; Garrett et al., 2012; Lee & Lee, 2014). Gaze patterns on corresponding areas of interest (AOIs) showed differential dwell times of the three groups. Specifically, both trauma-exposed groups (PTSD, TEHC) dwelled longer on negative-valence over neutral faces, with a greater bias noted in the PTSD group than in the TEHC group. Conversely, the HC group showed the opposite pattern, dwelling longer on neutral stimuli over negatively-valenced faces. These findings extend considerable other work with this eye-gaze paradigm (e.g. Abend et al., 2020; Chong & Meyer, 2020; Klawohn et al., 2020; Lazarov et al., 2021a; Lazarov, Abend, & Bar-Haim, 2016; Lazarov, Ben-Zion, Shamai, Pine, & Bar-Haim, 2018), establishing sustained attention as an important cognitive feature of emotional problems, including in PTSD.

Previous neuroimaging research has identified several brain regions as involved in attention processes. Studies examining attention allocation toward/away from threat stimuli in healthy individuals implicate frontolimbic circuitry encompassing the amygdala, anterior cingulate, and areas in the prefrontal cortex (PFC), especially the lateral and medial parts (Bishop, 2008; Shechner et al., 2012; Shechner & Bar-Haim, 2016). Corresponding perturbations in the function of these areas have also been found in attentional research among anxious individuals (Britton et al., 2012, 2015; Eldar & Bar-Haim, 2010; Fani et al., 2012; Monk et al., 2008; Taylor et al., 2014; Telzer et al., 2008). Finally, altered activity of the striatum, a reward-related brain region, has been also implicated in attentional tasks among anxious individuals (Shechner et al., 2012). Interestingly, neuroimaging studies focusing on PTSD in general show perturbations in similar brain regions, noted above, implicating the amygdala, the dorsal anterior cingulate cortex (dACC), and the lateral and medial PFC (for a review, see Hayes, Hayes, & Mikedis, 2012a; Hayes, Vanelzakker, & Shin, 2012b), as well as altered activity in the nucleus accumbens (NAcc), a key component of the reward system (Admon et al., 2013; Boukezzi et al., 2020; Elman et al., 2009; Pessin, Philippi, Reyna, Buggar, & Bruce, 2021; Sailer et al., 2008). Lastly, and most relevant for the present study, all of the above-cited areas have been also specifically implicated in functional connectivity studies in PTSD (Koch et al., 2016; Kunimatsu, Yasaka, Akai, Kunimatsu, & Abe, 2020; Pessin et al., 2021; Zhu et al., 2016).

The aim of the present study was to explore the neural correlates of sustained attention found in the above-cited attentional study of PTSD (Lazarov et al., 2021b). To that end, we used rsFC MRI data and attention allocation indices of a sub-sample of participants of the original study, who provided both types of data (Lazarov et al., 2021b). We employed an exploratory analytic approach as the neural correlates of sustained attention, measured via a free-viewing eye-tracking-based paradigm, have not been studied before. In line with the above-reviewed literature, we explored whether group differences in attention allocation might be related to different rsFC between these primary regions of interest [ROIs; amygdala, dACC, dorsolateral prefrontal cortex (dlPFC), ventrolateral prefrontal cortex (vlPFC), and NAcc], implicated in prior research on both PTSD and attention. While exploratory, based on our previous findings showing group differences in sustained attention on threat (*v.* neutral stimuli), we expected to find corresponding group differences in the rsFC-to-DT% associations of brain areas involved both in threat-related attention allocation (Bishop, 2008; Shechner et al., 2012; Shechner & Bar-Haim, 2016) and in PTSD (for a review, see Hayes et al., 2012a, 2012b; Suarez-Jimenez et al., 2020), namely, the amygdala, dACC, dlPFC, or vlPFC.

## Methods

### Participants

Participants were a sub-sample of participants who took part in the above-described study on attention allocation in PTSD (see Lazarov et al., 2021b for a full description of the original sample and the recruitment methods). Specifically, included participants were those who were found to be eligible for, and agreed to participate in, a subsequent MRI study conducted the same day. Participants of the original sample that were not MRI-eligible or that declined participation in the subsequent MRI study were not included in the present study. The final (sub)sample included 17 participants with PTSD, 21 TEHCs, and 16 HCs with no trauma exposure, matched on age, sex, and race. Demographic and psychopathological characteristics by group are presented in Table 1. See online Supplementary Material for a detailed group differences analyses on these characteristics.

**Table 1.** Demographic and psychopathological characteristics by group

	PTSD group (*n* = 17)		TEHC group (*n* = 21)		HC group (*n* = 16)	
	*M*	s.d.	*M*	s.d.	*M*	s.d.
Age	45.71	15.32	37.05	12.24	37.13	10.30
Gender ratio (M:W)	9:8	–	13:8	–	7:9	–
Race (% white)	88.23	–	76.19	–	70.59	–
Education (years)*	13.94^a^	2.11	15.29^b^	1.93	16.56^b^	2.94
Age at trauma	30.47	15.20	26.29	11.02	–	–
Time since trauma	15.24	12.36	10.76	10.01	–	–
CAPS*	33.71^a^	7.04	1.43^b^	2.40	–	–
HAM-D*	14.35^a^	4.72	1.05^b^	1.60	0.25^b^	0.68
HAM-A*	27.24^a^	40.15	1.57^b^	3.75	0.19^b^	0.55

PTSD, posttraumatic stress disorder; TEHC, trauma exposed healthy control; HC, healthy control; CAPS, Clinician-Administered PTSD Scale; HAM-D, Hamilton Rating Scale for Depression; HAM-A, Hamilton Anxiety Rating Scale.

*Note.* Asterisks indicate significant differences between groups. Different superscripts indicate significant pair-wise differences between groups.

All trauma-exposed participants (PTSD, TEHC) met DSM-5 criterion A for a traumatic event of an interpersonal nature, determined using the Life Events Checklist for DSM-5 (LEC-5; Weathers et al., 2013). In addition to a primary diagnosis of PTSD, participants with PTSD also scored ⩾25 on the Clinician-Administered PTSD Scale-5 (CAPS-5; Weathers et al., 2013, see below). TEHCs had no current/past diagnosis of PTSD coupled with a CAPS-5 score <10. HCs had no current/past diagnosis of any psychiatric disorder. See online Supplementary Material for detailed inclusion/exclusion criteria.

The study adhered with the ethical guidelines of the Declaration of Helsinki and was approved by the New York State Psychiatric Institute (NYSPI) Institutional Review Board (Protocol #7464: visual attention in PTSD). After receiving explanations about the study, participants provided written informed consent.

### Measures

Primary and co-morbid psychiatric diagnoses were assessed by an independent clinical assessor, a PhD-level psychologist trained to 85% reliability with a senior clinician, using the Structured Clinical Interview for DSM-5 (SCID-5; First, Williams, Karg, & Spitzer, 2015) – an established and validated interview for DSM-5 diagnoses. Clinician-rated depression and anxiety symptoms were assessed using the clinician-rated Hamilton Rating Scale for Depression (HAM-D; Hamilton, 1960) and Hamilton Anxiety Rating Scale (HAM-A; Hamilton, 1959), respectively. Trauma-exposed participants (i.e. PTSD and TEHC participants) also completed the LEC-5 (Weathers et al., 2013) to assess trauma exposure, with CAPS-5 (Weathers et al., 2013) used to determine severity of symptoms in reference to each participant's identified traumatic event (on the LEC-5) as bothering them the most. See online Supplementary Material for a full description of all used measures.

### Free viewing eye-tracking task

Eye movements were gauged using a free-viewing eye-tracking task shown to have satisfactory psychometric properties in prior research of attention allocation in both depression and anxiety (Chong & Meyer, 2020; Klawohn et al., 2020; Lazarov et al., 2016, 2018, 2021a), and also specifically in PTSD (Lazarov et al., 2021b). The task was designed and implemented using the Experiment Builder software (version 2.1.140; SR Research Ltd., Mississauga, Ontario, Canada).

**Fig. 1.**
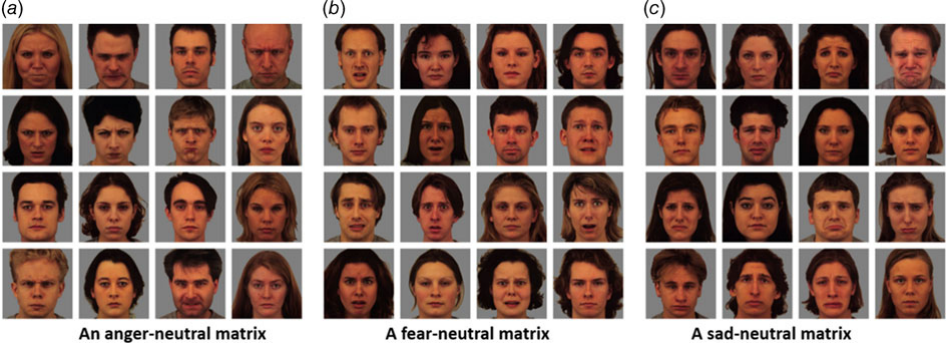
An example of a single matrix for (a) the anger-neutral block; (b) the fear-neutral block; and (c) the sad-neutral block. In each block the eight emotional faces comprise the anger/fear/sad area of interest (AOI) and the eight neutral faces comprise the neutral AOI.

Three separated blocks were included in the task, with each block focusing on a specific negative emotion-neutral contrast with theoretical relevance for PTSD – anger and neutral facial expressions, fear and neutral facial expressions, and sad and neutral facial expressions. Blocks were counterbalanced across participants within each group. Stimuli for each block was assembled using chromatic photographs of eight male and eight female actors, each contributing an emotional and a neutral facial expression, for a total of 32 pictures (16 male and 16 female), with each actor appearing in only one of the three blocks. Pictorial faces were taken from the Karolinska Directed Emotional Faces database (KDEF; Lundqvist, Flykt, & Öhman, 1998). Each experimental block comprised of 30 different face matrices. Each matrix consisted of 16 faces, eight negative emotional faces (anger, fear, or sad) and eight neutral facial expressions, arranged in a 4-by-4 layout. Single faces were 225-by-225 pixels in size, including a 10-pixel white margin frame, for an overall matrix size of 900-by-900 pixels (see Fig. 1 for a matrix example of each block). Location of single faces within the matrix was determined randomly while adhering with three guidelines: (a) each actor appeared only once in a matrix; (b) half of the faces were male and half female; and (c) half the faces were emotional and half were neutral, a ratio kept also by the four inner faces of the matrix. Finally, each single facial expression appeared exactly 15 times per block

Each trial of the task was comprised of a fixation cross mandating a 1 s fixation for the trial to proceed, followed by 6 s of freely viewing the matrix, and ending with a 2 s inter-trial-interval. A five-point eye-tracking calibration followed by a five-point validation procedure preceded each block, necessitating a visual deviation below 0.5° on all points, on both the *X* and *Y* axes. A 2 min break was introduced between blocks to reduce fatigue.

### Eye-tracking measures

Eye-tracking data were processed using EyeLink Data Viewer software (version 3.1.246; SR Research Ltd.). Fixations were defined as at least 100 ms of stable fixation within 1-degree visual angle. For each presented matrix we defined two AOIs, one including the eight negatively-valenced faces (anger, fear, or sad; the negative AOI) and one including the eight neutral faces (the neutral AOI). Attention allocation was quantified as *percent dwell time* on the emotional AOI (DT%) in each block, namely, the total dwell time on the emotional AOI out of the total dwell time on both AOIs (emotional + neutral faces; Lazarov et al., 2016, 2018, 2021b; Lazarov, Pine, & Bar-Haim, 2017), an attentional measure showing good psychometric properties in PTSD (Lazarov et al., 2021b).

### Apparatus

A remote high-speed eye tracker (Eyelink 1000+; SR Research Ltd.) was used to record eye movements, at a 500 Hz sampling rate. Distance between participants' eyes and the eye-tracking monitor was 60-to-65 cm. We used a 24-inch monitor with a screen resolution of 1920 × 1080 pixels.

### Neuroimaging data acquisition and imaging data preprocessing

Of the 54 participants that provided rsFC data, 16 were scanned using a 3 T General Electric MR750 (PTSD = 7, TEHC = 4, HC = 5), and 38 (PTSD = 10, TEHC = 17, HC = 11) using a 3 T General Electric PREMIER (GE Medical Systems, Waukesha, WI, USA) due to scanner upgrade in NYSPI's MRI center. A 32-channel receive-only head coil was used. For each participant we collected a structural T1 and a resting state T2 scan. For each participant a high-resolution T1-weighted 3D BRAVO sequence was acquired using the following parameter: T1 = 450 mm, flip angle = 12°, field of view = 25.6 cm, 256 × 256 matrix, slice thickness = 1 mm. T2*-weighted echo-planar images depicting the blood-oxygen-level-dependent (BOLD) were acquired for 17 participants with repetition time (TR) = 1.3 s, echo time (TE) = 28 ms, flip angle (FA) = 60°, field of view (FOV) = 19.2 cm, number of slices = 27, slice thickness = 4 mm. Thirty-eight participants' BOLD were scanned with TR = 2 s, TE = 30 ms, FA = 90°, FOV = 24 cm, number of slices = 34, slice thickness = 3.5 mm. Participants were instructed to relax, remain awake, and lie still with their eyes open. A head cushion was used to limit head motion. Scans were conducted for 10 min.

All MRI images were preprocessed using MATLAB version R2018a (The MathWorks, Inc., Natick, MA, USA) and statistical parametric mapping software (SPM12; Welcome Trust Centre for Neuroimaging, UCL, London, UK). Three functional datasets were analyzed separately, using the same standard pipeline: first, slice-time corrected and motion corrected using a six-parameter rigid body transformation, then co-registered to each participant's T1-weighted structural image. Co-registered images were normalized to the Montreal Neurological Institute (MNI) canonical template, and smoothed with an 8 mm full-width-at-half-maximum Gaussian kernel. Functional connectivity analyses were performed on the smoothed images. We controlled for different scanners in all rsFC analyses.

### Procedure

Participants were tested individually at the Anxiety Disorders Clinic, NYSPI. They were told that they are going to participate in an eye-tracking study examining gaze patterns. After providing informed consent, rsFC was collected as described above. Following completion of the rsFC collection, participants were taken to the eye-tracking room. They were seated in front of the eye-tracking monitor and told that during the task they would be presented with different matrices of faces, appearing one after the other. They were also told that before the appearance of each matrix a fixation cross will appear at the center of the screen, on which they should fixate to make the matrix itself appear. Participants were instructed to look freely at each matrix until it disappeared.

### Data analysis

#### Demographics and psychopathological characteristics

One-way analyses of variance (ANOVAs) compared between-groups descriptive characteristics, including HAM-D and HAM-A scores, with χ^2^ tests used to compare groups on gender and ethnicity ratios. An independent sample *t* test was also used to compare the trauma-exposed groups on CAPS-5 scores. Follow-up analyses for significant one-way ANOVAs included independent sample *t* tests and χ^2^ tests for gender ratio and ethnicity. See online Supplementary Material for results of these analyses.

#### Eye-tracking data

We examined group differences on the eye-tracking measures by performing a three-by-two mixed-model ANOVAs with group (PTSD, TEHC, HC) as a between-subjects factor and emotional block (anger, fear, sad) as a within-subject factor. Because the three groups differed in the number of years of education, this variable was introduced as a covariate in all analyses.

Statistical analyses were conducted using SPSS (IBM; version 25.0) and were two-sided, using *α* of 0.05. Effect sizes are reported using 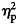 values for ANOVAs and Cohen's *d* for mean comparisons.

#### Seed-based functional connectivity analyses

rsFC analyses were carried out using a seed-based approach implemented in the CONN-MRI Functional Connectivity toolbox v13. Band-pass filtering with a frequency window of 0.01–0.09 Hz was performed. Outlier detection was carried out with artifact detection tools implemented in CONN. The principal component-based noise-correction method, ‘CompCor’, implemented in this toolbox, was used for additional control of physiological noise and head motion effects. Outlier volumes in each participant were identified as having large spiking artifacts (i.e. volumes >3 standard deviations from the mean image intensity), or large motion (i.e. 0.5 mm for scan-to-scan head-motion composite changes in the *x*, *y*, or *z* direction). Anatomical images were segmented into grey matter, white matter, and cerebrospinal fluid (CSF) regions. Covariates corresponding to head motion (six realignment parameters and their derivatives), outliers (one covariate per outlier consisting of 0s everywhere and a 1 for the outlier time point), and the BOLD time series from the subject-specific white matter and CSF masks were used in the connectivity analysis as predictors of no interest, and were removed from the BOLD functional time series using linear regression. Global signal was not used as nuisance variable due to the potential bias that can be introduced (Murphy & Fox, 2017).

No participant met the movement exclusion criteria (exceeding ±1.5 mm, and the fact that more than 20% of their data points having been detected as outliers), thus all participants were entered into the final analysis. No significant difference in head motion was found among the three groups (*p* > 0.5).

ROI-to-whole-brain voxel connectivity analysis was performed using seed ROIs identified as key regions in networks implicated in PTSD including the amygdala, dACC, dlPFC, vlPFC, and NAcc. All ROIs were derived from the AAL atlas or Brodmann areas. DlPFC covers BA 9, 10, and 46; VlPFC covers BA 11, 44, 45, 47; and dACC covers BA32. Next, five seed ROI-to-voxel brain analyses were carried out. In the subject-level analysis, bivariate-regression analyses were used to determine the linear association of the BOLD time series between each seed ROI and all other voxels within the brain for each subject. Both positive and negative correlations were examined. The resultant correlation coefficients were transformed into *z* scores using Fisher's transformation to satisfy normality assumptions. To examine the association between the functional connectivity and the percentage dwell time (DT%) on threat faces among the three groups, a group-level general linear model was used with DT% as a dependent variable, brain connectivity as an independent variable, and group as a fixed factor. Seed-to-whole brain connectivity maps for all five seed ROIs were examined and tested for seed-to-voxel connectivity correlation with DT% across the brain while controlling for education and scanner. Therefore, education and scanner were regressed out as covariate of no interests. The DT% and covariate of no interests (education, scanner) were demeaned before entering the regression analysis. An FDR-corrected significance threshold of *p* < 0.05 was used. To test the direction of the activation, Marsbar toolbox (marsbar.sourceforge.net) was used to extract the ROI connectivity weights. For the NAcc-OFC post-hoc analysis, the residualized correlation weight was used, with scanner and education regressed out during the whole brain analysis.

## Results

### Attention allocation (percent dwell time on threat): replication analysis

Results mostly replicated the findings of the original study with the full sample (Lazarov et al., 2021b). While the group × emotional block interaction was not significant [*F*_(2, 50)_ = 0.93, *p* = 0.40], a significant main effect of group emerged [*F*_(2, 50)_ = 5.13, *p* = 0.009, *η^2^_p_* = 0.17], indicating group differences on DT% across the three blocks. No significant main effect was noted for emotional block [*F*_(1, 50)_ = 0.10, *p* = 0.75]. We therefore collapsed across blocks for the between-groups follow-up analyses, by computing the percent dwell time (DT%) on the negative-valenced AOI – the total dwell time on the anger, fear, and sad AOIs, out of the total dwell time on the negative-valenced and the neutral-valenced AOI (i.e. the total dwell time on the neutral AOI from each of the three blocks). Results showed a significant group difference between the PTSD (*M* = 0.54, s.d. = 0.04) and HC (*M* = 0.50, s.d. = 0.03) groups [*t*(31) = 2.85, *p* = 0.008, Cohen's *d* = 1.13], which also emerged for the TEHC (*M* = 0.53, s.d. = 0.03) and HC groups [*t*(35) = 2.30, *p* = 0.03, Cohen's *d* = 1.00]. The TEHC and PTSD groups did not differ on DT% [*t*(36) = 0.97, *p* = 0.34].

### Neural correlates of attention allocation: associations with rsFC

**Fig. 2.**
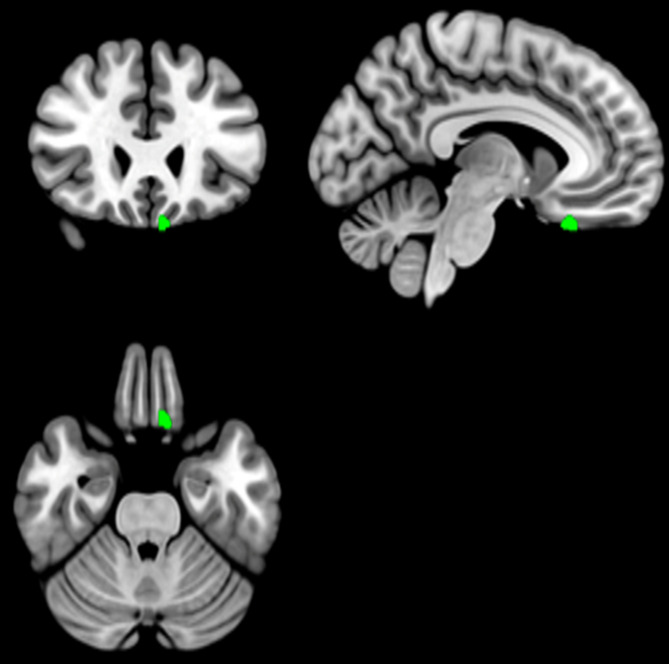
The NAcc-OFC pathway [−9, 24, −28].

**Fig. 3.**
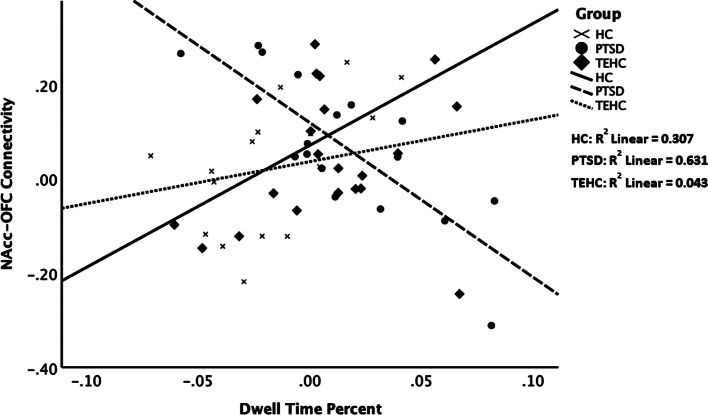
NAcc-OFC resting-state connectivity associated with (demeaned) dwell time percent (DT%) on negative-valenced stimuli by group. NAcc, nucleus accumbens; OFC, orbitofrontal cortex; HC, healthy controls; PTSD, posttraumatic stress disorder; TEHC, trauma-exposed healthy control.

Results showed that the NAcc-OFC pathway-to-DT% (see Fig. 2) was the only significant association among the examined seed ROI maps to whole-brain voxel-wise connectivity that survived the FDR correction (*p* < 0.05). To clarify the direction of this association, we extracted the ROI values within the OFC, and plotted against DT% for the three groups (see Fig. 3). While in the PTSD group results revealed a significant negative NAcc-OFC pathway-to-DT% association [*r*(17) = −0.79, *p* < 0.001], a significant positive NAcc-OFC pathway-to-DT% association emerged for the HC group [*r*(16) = 0.58, *p* = 0.02]. For the TEHC group, a non-significant positive association was noted [*r*(21) = 0.21, *p* = 0.36].

## Discussion

The current exploratory study examined whether attention allocation to negative-valence and neutral faces during a free-viewing eye-tracking task relates to rsFC patterns in three groups of participants – patients with PTSD, trauma-exposed healthy control participants with no PTSD, and healthy participants with no prior exposure to a traumatic event. While mostly replicating the eye-tracking-based attentional results of the larger sample of the original study (Lazarov et al., 2021b)^†^^1^, from which current study participants were drawn, the current study also extends previous findings by exploring the neural correlates associated with emergent attentional processes. Specifically, significant group differences emerged on sustained attention between the two trauma-exposed groups (i.e. PTSD, TEHC) and the HC group, while the TEHC and PTSD groups did not differ. In contrast to differentiating participants based on trauma-exposure (as PTSD and TEHC participants did not differ on attention allocation), the NAcc-OFC neural connectivity marker appeared to dissociate participants along the symptoms dimension. Here, for patients with PTSD, but neither the TEHC or HC groups, attention allocation to negative-valance stimuli inversely correlated with connectivity. In fact, in the HC group this correlation was significantly positive.

Considering group differences on attention allocation to negatively-valenced stimuli concurrently with the groups' NAcc-OFC pathway-to-DT% associations may shed some light on the possible relationship between attention allocation and related neural activation patterns. While the PTSD and TEHC groups did not differ on attention allocation to negative-valenced stimuli, with both groups showing increased DT% compared to the HC group, the TEHC group was more similar to the HC group on the NAcc-OFC-to-DT% association, with both groups showing a positive association, not a negative one characterizing the PTSD group. While exploratory in nature, this may represent a possible resilience factor in TEHC participants emanating from sufficient recruitment of reward-related pathways enabling positive affect regulation when confronted with negative-valenced stimuli. Surprisingly, and contrary to our expectations, we did not find any group differences in rsFC-to-DT% associations in other brain regions such as the amygdala, dACC, dlPFC, or vlPFC – areas involved in threat-related attention allocation (Bishop, 2008; Shechner et al., 2012; Shechner & Bar-Haim, 2016), found to be aberrant in anxious individuals (Britton et al., 2012, 2015; Eldar & Bar-Haim, 2010; Fani et al., 2012; Monk et al., 2008; Taylor et al., 2014; Telzer et al., 2008), including those with PTSD (for a review, see Hayes et al., 2012a, 2012b; Suarez-Jimenez et al., 2020). While this lack of findings may be related to the small sample size in the current study (see limitations section below), it may also reflect the possibility that all groups are indeed not different in their rsFC-to-DT% associations in these areas. Put differently, it might be that the ability to disengage negatively-valenced stimuli is less related to brain areas of threat-related attention modulation, but rather to a reward-related network including the NAcc-OFC pathway. This finding is in line with extant literature documenting reduced reward-related functional connectivity in PTSD (for a recent review see Neria, 2021), particularly in pathways associated with the NAcc and OFC. Still, the current study is the first to link NAcc-OFC connectivity to attention modulation of negatively-valenced stimuli. The association between the ability to disengage negatively-valenced stimuli with the strength of the NAcc-OFC pathway connectivity found in the control groups may contribute to our understanding of resilience in the face of exposure to trauma.

The finding is notable concerning attention allocation and connectivity of the NAcc-OFC pathway among patients with PTSD. Both the OFC and NAcc are considered specific neuroanatomical areas within the reward circuit, underlying reward processing (Castro & Bruchas, 2019; Dillon et al., 2008; Goto & Grace, 2008; Kringelbach, 2005; Mannella, Gurney, & Baldassarre, 2013; Ng, Alloy, & Smith, 2019; Rizvi, Pizzagalli, Sproule, & Kennedy, 2016). Altered activity of both these areas has been implicated in aberrant reward processing, as evident in anhedonia and MDD (for reviews see Ng et al., 2019; Rizvi et al., 2016), and with dysregulated corticostriatal connectivity (i.e. OFC-to-NAcc connectivity), which has been specifically highlighted in this regard (for reviews see Admon & Pizzagalli, 2015; Ng et al., 2019). Dysregulated corticostriatal connectivity was further associated with a specific inability to sustain and regulate positive affect (Heller et al., 2009). Based on these findings, one may interpret a positive association between sustained attention on negative-valance stimuli and NAcc-OFC connectivity, noted in the HC group, as signaling the recruitment of a top-down reward-related pathway (the NAcc-OFC pathway), enabling affect regulation when confronted with negative-valenced stimuli. Conversely, in the PTSD group, a negative association emerged, reflecting disordered affect regulation in the wake of negative-valenced stimuli, which may lead to sustained attention on these stimuli. This exploratory finding corresponds with approaches viewing PTSD as an imbalance of the negative (fear-related) and positive (reward-related) valence systems, with the former attaining precedence over the latter (Admon et al., 2013; Stein & Paulus, 2009).

Several limitations of this exploratory study should be noted, which warrants further investigation. First, the current samples were relatively small; therefore, the analyses and interpretations of current results should be taken with caution. Given larger samples, significant group differences on attention allocation to threat between the PTSD and TEHC groups might have also been noted, as was the case in original study with the larger samples (Lazarov et al., 2021b). Additionally, with larger sample, other functional connectivity patterns might have emerged, for instance between the applied seeds and attention- or threat-related brain areas. The salience network (SN) has been attributed with the detection and filtering of salient stimuli, such as negative-valence stimuli, to generate appropriate behavioral responses (Menon & Uddin, 2010). In PTSD, overactive SN areas, such as the insula and dACC, have been attributed with the destabilization of other brain networks (Akiki, Averill, & Abdallah, 2017), and in so with the destabilization of appropriate threat-response behavior. Therefore, a bigger sample size could have yielded significant difference among the groups in the SN-to-DT% association. Finally, given a larger sample size, we might have had the power to do whole-brain analysis which may have yielded significant results in areas we did not assess in the present study. Second, the present study examined attention allocation patterns to negatively-valenced faces, and did not include emotional blocks contrasting neutral/negatively-valenced stimuli with positively-valenced ones. As present findings associate reward-related brain areas with attention allocation to negatively-valenced stimuli, future studies should examine attention allocation while incorporating positive-valence stimuli, to better probe this unexpected finding. Third, the present study used negatively-valenced faces rather than trauma-specific stimuli, which could elicit other connectivity patterns related to trauma. However, as we only included participants for whom DSM-5 criterion A was of an interpersonal nature, we believe that faces are highly relevant stimuli for this cohort (Armstrong et al., 2013; Fonzo et al., 2010; Garrett et al., 2012; Lee & Lee, 2014). Finally, this study focused on picture-based stimuli. Using more complex stimuli, such as videos or virtual reality tasks, to explore attention allocation could elicit stronger or different brain reactions. Future studies could utilize novel technologies to assess attentional patterns in more complex threatening situations.

In conclusion, the current exploratory study suggests that reward-related brain areas might be influential in disengaging from negative-valenced stimuli, and possibly regulating ensuing emotions. Thus, harnessing the reward system and reward-related attentional processes could augment extant attention training procedures for PTSD. Extant attention training procedures in PTSD have all exclusively targeted aberrant attention patterns using contrasting threat and neutral stimuli (e.g. threat *v.* neutral words, anger *v.* neutral faces, or trauma-related *v.* neutral pictures; Alon, Azriel, Pine, & Bar-Haim, 2022; Badura-Brack et al., 2015; Kuckertz et al., 2014; Lazarov et al., 2019a, 2019b; Niles et al., 2020; Schoorl, Putman, & van Der Does, 2013; Segal, Pine, & Bar-Haim, 2020), with no study to date exploring the clinical efficacy of training procedures contrasting positive/rewarding stimuli (e.g. positive words, happy faces, or positive pictures) with either threat or neutral ones. Future research could also utilize more recent and advance gaze-contingent reward-based procedures in which positive reinforcement is used to encourage more adaptive attentional allocation processes (Lazarov et al., 2017; Shamai-Leshem, Lazarov, Pine, & Bar-Haim, 2021).

## Footnotes

† The notes appear after the main text.

## Note

1 While in the original study (Lazarov et al., 2021a, 2021b) we examined dwell time per area of interest (AOI), including AOI (i.e. negative-valence, neutral) as another within-subject variable, here, to correlate attention allocation with rsFC we chose to measure attention allocation using the percent dwell time measure, a measure we have used repeatedly in previous studies using the same task when needing to correlate attention allocation with other measures. Re-analyzing the original data set using the DT% measure did not change the original results pattern – significant group differences between the PTSD and TEHC groups, the PTSD and HC groups, and the TEHC and HC groups.

## Supplementary material

For supplementary material accompanying this paper visit https://doi.org/10.1017/S003329172200157X. click here to view supplementary material
