# Supplementary material for: Attention allocation to negatively-valenced stimuli in PTSD is associated with reward-related neural pathways
Source: Psychol Med. 2022 Jun 2;53(10):4666–74. doi: 10.1017/S003329172200157X (PMC9715854; doi:10.1017/S003329172200157X)
Supplement: Supplementary file 1 [file S003329172200157Xsup001.docx]

**Supplemental Material**

**Methods**

**Participants**

**Demographics and psychopathological characteristics**

Demographic and clinical characteristics of the three groups are described in Table 1. As expected, a significant difference between the PTSD and TEHC groups was noted for CAPS-5 scores, *t*(36)=19.69, p<.001, *Cohen’s d*=6.14. Significant group differences (PTSD, TEHC, HC) were noted for HAM-D depression scores, *F*(2, 53)=134.09, *p*<.001, *η^2^_p_* =.84, and for HAM-A anxiety scores, *F*(2, 53)=7.87, *p*=.001, *η^2^_p_* =.24. Follow-up analyses revealed a higher HAM-D depression score in the PTSD group compared with both the TEHC group, *t*(36)=12.13, *p*<.001, *Cohen's d*=3.78, and the HC group, *t*(31)=11.83, *p*<.001, *Cohen's d*=4.18. This pattern also emerged for the anxiety score, *t*(36)=2.92, *p*=.006, *Cohen's d*=0.90, and *t*(31)=2.69, p=.01, *Cohen's d*=0.95, respectively. The TEHC group and HC groups did not differ on neither HAM-D scores, *t*(35)=1.87, *p*=.07, nor HAM-A scores, *t*(35)=1.46, *p*=.15. Significant differences between groups were also noted for education, *F*(2, 53)=5.26, *p*=.008, *η^2^_p_* =.17, with a lower average in the PTSD group compared with both the HC, *t*(31)=2.96, *p*=.006, *Cohen's d*=1.04, and the TEHC groups, *t*(36)=2.05, *p*=.04, *Cohen's d*=0.67, which did not differ on education, *t*(35)=1.59, *p*=.12. Finally, no group differences were noted for age, *F*(2, 53)=2.65, *p*=.08, Gender ratio, *χ^2^*(2)=1.21, *p*=.54, Race, *χ^2^*(4)=3.31, *p*=.51, age at trauma, *t*(36)=0.98, *p*=.33, or time that passed since the traumatic event, *t*(36)=1.23, *p*=.23.

**Detailed Inclusion and Exclusion Criteria**

Inclusion criteria for the PTSD group were: a) primary diagnosis of PTSD; b) CAPS-5 score >25; c) 18-80 years of age; and d) fluency in English. Exclusion criteria were: a) current severe depression indicated by a HAM-D (Hamilton, 1960) score ≥25 (Zimmerman, Martinez, Young, Chelminski, & Dalrymple, 2013); b) suicidal ideation or behavior; c) current or history of psychosis; d) current or past diagnosis of OCD, bipolar disorder, manic episode, tic disorder, or ADHD; e) current or past organic mental disorder, seizure disorder, epilepsy or brain injury; f) diagnosis of probable Alzheimer’s disease, Vascular Dementia or Parkinson’s disease; g) current unstable or untreated medical illness; h) drug or alcohol misuse - severe alcohol/cannabis disorder or any other substance use disorder except nicotine; i) recent psychotropic medication change or initiation within the last 3 months; j) initiation of psychotherapy within the last 3 months; k) Current cognitive impairments as a result of a traumatic brain injury (TBI); and l) eye-tracking calibration difficulties. Additional exclusion criteria related to rsFC collection were: m) any condition that would exclude an MRI exam (e.g., pacemaker, paramagnetic metallic implants or devices, any other non-removable paramagnetic metal in the body); n) significant claustrophobia that would preclude ability to remain calm within the MRI scanner; and o) left handedness. Of the 17 participants with PTSD included in the study, 11 also met criteria for current MDD or persistent depressive disorder (PDD), four for generalized anxiety disorder (GAD), one for panic disorder, two for social anxiety disorder (SAD), and two for eating disorders (ED).

Inclusion criteria for the TEHC group were: a) experiencing a Criterion A traumatic event of an interpersonal nature; b) 18-80 years of age; and c) fluency in English. Exclusion criteria were: a) present or past PTSD diagnosis; b) significant PTSD symptoms indicated by a CAPS-5 score >10; c) depressive symptoms as indicate by a HAM-D score >8; and d) suicidal ideation or behavior. Additional exclusion criteria were criteria c-to-o as detailed above for the PTSD group. None of the TEHC participants had a current psychiatric diagnosis. One had past diagnosis of MDD.

Inclusion criteria for the HC group were: a) 18-80 years of age; and b) fluency in English. Exclusion criteria were: a) current or past history of any DSM-5 psychiatric disorder; b) experience of a traumatic event (of any kind) or events in childhood and/or adulthood adhering to DSM-5 Criterion A for PTSD; c) depressive symptoms as indicate by a HAM-D score >8; and d) suicidal ideation or behavior. Additional exclusion criteria were criteria e-to-o as detailed above for the PTSD group.

We only invited participants that had normal or corrected-to-normal vision, excluding usage of multi-focal eye wear to prevent eye-tracking calibration difficulties.

**Measures**

**Trauma exposure**

Trauma exposure was determined using the Life Events Checklist for DSM-5 (LEC-5; Weathers et al., 2013)), an updated version of the original psychometrically-sound LEC (Gray, Litz, Hsu, & Lombardo, 2004). It is a 17-item self-report measure assessing exposure to potentially traumatic events (e.g., physical assault, sexual assault, combat) during one’s life time. For each event participants are required to indicate if they experienced the event personally, witnessed it, learned about it, experienced it as part of their job, were not sure if they experienced it, or felt the event did not apply to them. From the events reported, participants are asked to identify the one event that currently bothers them the most and reference this event when completing the PTSD-related measures.

**Clinician-rated PTSD**

Severity of PTSD symptoms was measured using CAPS-5 (Weathers et al., 2013). The CAPS-5 is a structured interview diagnosing PTSD based on DSM-5 criteria. It has been widely used in research demonstrating excellent reliability, convergent and discriminant validity, diagnostic utility and sensitivity to change (Weathers et al., 2018; Weathers, Keane, & Davidson, 2001). Cronbach’s α in the current sample was .95.

**Clinician-rated Depression**

Clinician-rated depressive symptoms were measured using the HAM-D (Hamilton, 1960), a 17-item measure covering the core symptoms of depression as manifesting over the past week. The HAM-D has been shown to have strong internal consistency and inter-rater and test-retest reliability (Trajkovic et al., 2011). In the current study the HAM-D was administered using the Structured Interview Guide for the Hamilton Rating Scale for Depression (SIGH-D; Williams, 1988) shown to have strong psychometric properties in clinical samples (Williams, 1988). Cronbach’s α in the current sample was .90.

**Clinician-rated Anxiety**

Clinician-rated anxiety symptoms were measured using HAM-A (Hamilton, 1959), a 14-item questionnaire measuring anxiety symptoms over the past week. The HAM-A was administered using the Structured Interview Guide for the Hamilton Anxiety Rating Scale (SIGH-A; Shear et al., 2001), which has been shown to have higher inter-rater and test-retest reliability compared with the regular format (Shear et al., 2001). Cronbach’s α in the current sample was .93.

**References**

Gray, M. J., Litz, B. T., Hsu, J. L., & Lombardo, T. W. (2004). Psychometric properties of the Life Events Checklist. *Assessment, 11*(4), 330–341. https://doi.org/10.1177/1073191104269954

Hamilton, M. (1959). The assessment of anxiety states by rating. *British journal of medical psychology*, *32*(1), 50-55. doi:10.1111/j.2044-8341.1959.tb00467.x

Hamilton, M. (1960). A Rating Scale for Depression. *Journal of Neurology, Neurosurgery and Psychiatry, 23*(1), 56-62. doi:10.1136/jnnp.23.1.56

Shear, M. K., Vander Bilt, J., Rucci, P., Endicott, J., Lydiard, B., Otto, M. W., ... Frank, D. M. (2001). Reliability and validity of a structured interview guide for the Hamilton Anxiety Rating Scale (SIGH-A). *Depression and anxiety, 13*(4), 166-178. doi:10.1002/da.1033

Trajković, G., Starčević, V., Latas, M., Leštarević, M., Ille, T., Bukumirić, Z., & Marinković, J. (2011). Reliability of the Hamilton Rating Scale for Depression: a meta-analysis over a period of 49 years. *Psychiatry Research, 189*(1), 1-9. doi:10.1016/j.psychres.2010.12.007

Weathers, F. W., Blake, D. D., Schnurr, P. P., Kaloupek, D. G., Marx, B. P., & Keane, T. M. (2013). The clinician administered PTSD scale for DSM-5 (CAPS-5) [Assessment]. Retrieved from <http://www.ptsd.va.gov>

Weathers, F. W., Blake, D. D., Schnurr, P. P., Kaloupek, D. G., Marx, B. P., & Keane, T. M. (2013). Life Events Checklist for DSM-5 (LEC-5) [Assessment]. Retrieved from <http://www.ptsd.va.gov>

Weathers, F. W., Bovin, M. J., Lee, D. J., Sloan, D. M., Schnurr, P. P., Kaloupek, D. G., ... Marx, B. P. (2018). The Clinician-Administered PTSD Scale for DSM-5 (CAPS-5): Development and initial psychometric evaluation in military veterans. *Psychological assessment*, *30*(3), 383-395. doi:10.1037/pas0000486

Weathers, F. W., Keane, T. M., & Davidson, J. R. (2001). Clinician-administered PTSD scale: a review of the first ten years of research. *Depression and anxiety*, *13*(3), 132-156. doi:10.1002/da.1029

Williams, J. B. W. (1988). A Structured Interview Guide for the Hamilton Depression Rating Scale. *Archives of General Psychiatry, 45*(8), 742-747. doi:10.1001/archpsyc.1988.01800320058007

Zimmerman, M., Martinez, J. H., Young, D., Chelminski, I., & Dalrymple, K. (2013). Severity classification on the Hamilton depression rating scale. *Journal of Affective Disorders, 150*(2), 384-388. doi:10.1016/j.jad.2013.04.028
